# Prevalence and risk factors for depressive and anxiety symptoms in middle-aged Chinese women: a community-based cross-sectional study

**DOI:** 10.1186/s12905-022-01908-6

**Published:** 2022-07-29

**Authors:** Xueyin Wang, Gengli Zhao, Jiangli Di, Linhong Wang, Xiaosong Zhang

**Affiliations:** 1 Department of Obstetrics and Gynecology, Peking University First Hospital, No. 1 Xianmen Street, Xicheng District, Beijing, 100034 China; 2 Chinese Center for Disease Control and Prevention, National Centre for Women and Children’s Health, Beijing, China; 3 National Center for Chronic and Non-Communicable Disease Control and Prevention, Chinese Center for Disease Control and Prevention, Beijing, China

**Keywords:** Depressive symptoms, Anxiety symptoms, Menopause, Perimenopause, Postmenopause, Risk factors

## Abstract

**Background:**

Depression and anxiety have become main public health concerns globally. However, risk factors for depression and anxiety remain unclear. This study was to examine the prevalence and risk factors of depressive and anxiety symptoms in middle-aged Chinese women.

**Methods:**

This cross-sectional study, conducted in 2018, included 7,727 women aged 40–60 years from the eastern, central and western regions of China. Depressive and anxiety symptoms were determined by the Patient Health Questionnaire-9 and the Generalized Anxiety Disorders-7, respectively. Logistic regression models were used to estimate odds ratios (ORs) for depressive and anxiety symptoms in relation to sociodemographic, lifestyle and menopausal factors.

**Results:**

Among all participants, 19.5% (1 422/7 275) and 14.2% (1 035/7 275) of participants experienced depressive and anxiety symptoms, respectively. The multivariable logistic regression models showed that age, household income, regular physical activity, chronic diseases, menopausal status, vasomotor symptoms, somatic symptoms and urogenital symptoms were associated with depressive symptoms, while place of residence, regular physical activity, chronic diseases, vasomotor, somatic and urogenital symptoms were associated with anxiety symptoms.

**Conclusion:**

Depressive and anxiety symptoms were common among middle-aged Chinese women, and certain sociodemographic, lifestyle and menopausal symptoms have an important impact on the risk of depressive and anxiety symptoms.

## Background

Depression and anxiety are two major types of mental disorders, and have become prominent public health concerns globally [1, 2]. Women were more likely to suffer from both depressive and anxiety disorders than men from menarche onwards, and women may be at elevated risk of depression and anxiety during times of hormonal fluctuations including puberty, the pregnancy and postpartum periods, and the perimenopause stage [2–4]. It has been estimated that the prevalence of depressive and anxiety symptoms was 26.0% and 12.6% among Chinese women aged 40 to 60 years, respectively [5]. Previous evidence has indicated that symptoms of depression and anxiety are associated with increased risk of cardiovascular diseases, osteoporosis and osteoporotic fractures that substantially affected morbidity and mortality for middle-aged women [6–10]. In addition, both depressive and anxiety symptoms have a deleterious influence on women’s quality of life, sleep quality and life satisfaction during perimenopause [11–13].

Menopausal women have been proven to be susceptible to various psychological health problems, including depression and anxiety [14, 15]. Several menopausal symptoms, including hot flashes, night sweats, and insomnia, may also contributed to increased risk of depressive and anxiety symptoms [16–18]. A number of epidemiological studies have focused on influential factors of depressive and anxiety symptoms among middle-aged women with inconsistent findings. Several previous studies demonstrated that perimenopausal and postmenopausal women were more likely to report anxiety and depressive symptoms than premenopausal women [19–21], while another study did not observe significant association between menopausal status and symptoms of anxiety or depression [13]. An earlier study reported that higher body mass index (BMI) and single status were associated with higher risk of depressive symptoms among China’s aging population [22], whereas another cross-sectional study demonstrated BMI and marital status were not correlated to depressive symptoms in Chinese rural women [23]. Moreover, previous evidence mainly focused on the association of hot flashes and night sweats with symptoms of depression and anxiety [13], and did not elucidate the relationship between other menopausal symptoms (e.g. paresthesia, headache and urinary tract infection) and symptoms of depression and anxiety. Therefore, further research is needed to comprehensively explore the influential factors of depressive and anxiety symptoms among middle-aged women.

In this large-scale study, the validated instruments were utilized to assess depressive and anxiety symptoms and different domains of menopausal symptoms. The objective of this study was to evaluate the prevalence of depressive and anxiety symptoms according to menopausal status in middle-aged Chinese women, and further identify the association of sociodemographic, lifestyle and menopausal factors with depressive and anxiety symptoms.

## Methods

### Study design and participants

The National Survey of Women’s Health is a cross-sectional, community-based study of women residing in three socioeconomic regions of China: eastern (Jiangsu and Shandong provinces), central (Hunan and Anhui provinces), and western (Shanxi and Sichuan provinces). Details of this study have been described elsewhere [24]. Briefly, one urban and one rural areas were selected as investigation sites in each province, and we recruited women aged 10–70 years by a multi-stage stratified random cluster sampling at each investigation site. Face-to-face interviews were conducted to collect information on demographic characteristics, medical history, symptoms of depression and anxiety, menopausal symptoms (only collected in women aged ≥ 40 years) by using a structured questionnaire. Among 7,727 participants aged 40–60 years, the present analysis was limited to 7,275 participants. Those meeting the following criteria were excluded (n = 452): incomplete data collection for depressive symptoms (n = 84) or anxiety symptoms (n = 72); women who were in artificial menopause (n = 244); women who took estrogen in the past 6 months (n = 67); and women who had gynecological tumors (n = 78). This study was approved by the Ethical Review Committee of the Chinese Center for Disease Control and Prevention (ethic code: 201,810), and all participants provided a written informed consent.

### Assessment of depressive and anxiety symptoms

The Patient Health Questionnaire-9 (PHQ-9) was used to evaluate the presence of depressive symptoms during the past 14 days [25]. The Chinese version of the PHQ-9 has been used by several studies performed in Chinese populations, and has been proven to be adequate reliability and validity [26]. The PHQ-9 contains 9 items based on the diagnosis criteria of Diagnostic and Statistical Manual of Mental Disorders-IV, with a summed score ranging from 0 to 27. The answer categories were based on a 4-point response scale, with the categories ‘not at all’ (0), ‘various days’ (1), ‘more than half of the days’ (2) and ‘nearly every day’ (3). According to the sum of PHQ-9 scores, participants were classified into two groups: non-depressive symptoms (PHQ-9 score < 5) and depressive symptoms (PHQ-9 score ≥ 5) [27]. Cronbach’s α of the PHQ-9 was 0.86 in this study.

Anxiety symptoms were defined as a summed score of 5 or above based on the Chinese version of Generalized Anxiety Disorder (GAD-7) including 7 symptoms that had experienced during the past 14 days on a 21-point scale ranging from ‘not at all’ (0) to ‘nearly every day’ (3) [28]. According to the sum of GAD-7 scores, participants were classified into two groups: non-anxiety symptoms (GAD-7 score < 5) and anxiety symptoms (GAD-7 score ≥ 5) [29]. Cronbach’s α of the GAD-7 was 0.87 in this study.

### Assessment of independent factors

The interviewer-administered structured questionnaire was utilized to collect the information on socio-demographic characteristics (age, place of residence, education, employment status, marital status, average monthly household income and parity), lifestyle factors (drinking, smoking and regular physical activity), physical health factors (BMI and chronic diseases), and menopausal factors (menopausal status, vasomotor symptoms, somatic symptoms and urogenital symptoms). Regular physical activity was defined as women who engaged in physical activity at least 30 min per time, with no less than 3 times per week [30]. BMI was calculated as the weight in kilograms divided by the square of height measured in meters, and classified into four groups according to the Chinese standard [31]: underweight (BMI < 18.5 kg/m^2^), normal weight (18.5 kg/m^2^ ≤ BMI < 24 kg/m^2^), overweight (24 kg/m^2^ ≤ BMI < 28 kg/m^2^) and obese (BMI ≥ 28 kg/m^2^). Chronic diseases were defined as having at least one of the following diseases: hypertension, diabetes and dyslipidemia.

According to the 2011 Stages of Reproductive Aging Workshop + 10 criteria [32], menopausal status was categorized into (1) reproductive stage, defined as regular menstruation, or subtle changes in menstrual cycle characteristics; (2) perimenopause, defined as the beginning of a persist difference of 7 days in the length of consecutive cycles, or the last menstruation having occurred no more than 12 months; and (3) postmenopause, defined as the end of the 12 month period of amenorrhea.

The modified Kupperman Menopausal Index (KMI) in the Chinese version was used to assess the presence of menopause symptoms [33]. The KMI includes 13 items in four domains as follows: vasomotor (1 item), somatic (8 items), psychological (2 items), and urogenital (2 items) [34]. A scale ranging from 0 to 3 points is utilized to assess the severity of each item: 0, no symptoms; 1, mild symptoms; 2, moderate symptoms; and 3, severe symptoms. Due to the overlap between items in the KMI and those in the PHQ-9 or GAD-7, we excluded the 2 items in the psychological domain (mood swing and depression) and the 2 items (insomnia and fatigue) in the somatic domain from the KMI. Finally, the vasomotor domain included hot flashes and sweats; the somatic domain was composed of paresthesia, dizziness, arthralgia and myalgia, headache, palpitations, skin formication; the urogenital domain consisted of sexual problems and urinary tract infection. The total scores of vasomotor, somatic and urogenital symptoms were calculated by summing up all items included in each domain, respectively [35]. Participants were considered as having vasomotor, somatic or urogenital symptoms if they had the scores greater than or equal to the 75^th^ of the corresponding domain scores (vasomotor ≥ 1, somatic ≥ 3, urogenital ≥ 1).

### Statistical analysis

Demographic characteristics were presented as numbers and frequency distributions for categorical variables, or median and interquartile range for continuous variables, and were compared using the chi-square test or Mann–Whitney U test. We firstly performed univariate logistic regression models to assess the association of sociodemographic, lifestyle and menopausal factors with the presence of depressive or anxiety symptoms. Then, multivariable logistic regression models were conducted using variables associated with depressive or anxiety symptoms at the *P* < 0.1 level in each univariate model in order to evaluate the independent effect of these potential variables on depressive or anxiety symptoms. Analyses were conducted using SAS software version 9.4 (SAS Institute, Cary, NC). All *P* values are two-sided, and a 0.05 level was used to declare significant differences.

## Results

### Characteristics of study participants

Among 7,275 middle-aged women who met inclusion criteria, the proportions of reproductive stage, perimenopause and postmenopausal stage were 44.9%, 17.3% and 37.8%, respectively. Table 1 shows socio-demographic characteristics of participants in terms of menopausal status. Women in the reproductive stage were younger, and more likely to be well educated (13.4%), employed (84.9%), married (94.2%), and to have higher level of average monthly household income (4.7%), compared to those in the perimenopause and postmenopausal stage (all *P* < 0.001). The postmenopausal women were more likely to reside in urban areas (52.0%) and be multiparous (99.0%, both *P* < 0.001).

**Table 1:** Socio-demographic characteristics of participants by menopausal status

	Total	Reproductive stage	Perimenopause	Postmenopause	*P* value
Participants, n (%)	7275	3270 (44.9)	1257 (17.3)	2748 (37.8)	
Age, years, median (IQR)	48 (44, 52)	44 (42, 47)	47 (44, 49)	54 (50, 56)	< 0.001
Place of residence					< 0.001
Urban	3658 (50.3)	1659 (50.7)	570 (45.4)	1429 (52.0)	
Rural	3617 (49.7)	1611 (49.3)	687 (54.6)	1319 (48.0)	
Education					< 0.001
High school and below	6590 (90.6)	2833 (86.6)	1135 (90.3)	2622 (95.4)	
College or graduate school	685 (9.4)	437 (13.4)	122 (9.7)	126 (4.6)	
Employment status					< 0.001
Unemployed/retired	1403 (19.3)	494 (15.1)	206 (16.4)	703 (25.6)	
Employed	5872 (80.7)	2776 (84.9)	1051 (83.6)	2045 (74.4)	
Marital status					< 0.001
Married	6668 (91.7)	3080 (94.2)	1132 (90.1)	2456 (89.4)	
Single/divorced/widowed	607 (8.3)	190 (5.8)	125 (9.9)	292 (10.6)	
Average monthly household income, RMB					< 0.001
< 5000	4865 (66.9)	1932 (59.1)	851 (67.7)	2082 (75.8)	
5000–10,000	2114 (29.1)	1184 (36.2)	358 (28.5)	572 (20.8)	
> 10,000	296 (4.0)	154 (4.7)	48 (3.8)	94 (3.4)	
Parity					< 0.001
Nulliparous	134 (1.8)	77 (2.4)	31 (2.5)	26 (1.0)	
Multiparous	7141 (98.2)	3193 (97.6)	1226 (97.5)	2722 (99.0)	

Values are median (IOR) or n (%)

### The prevalence of depressive and anxiety symptoms by menopausal status

**Fig. 1:**
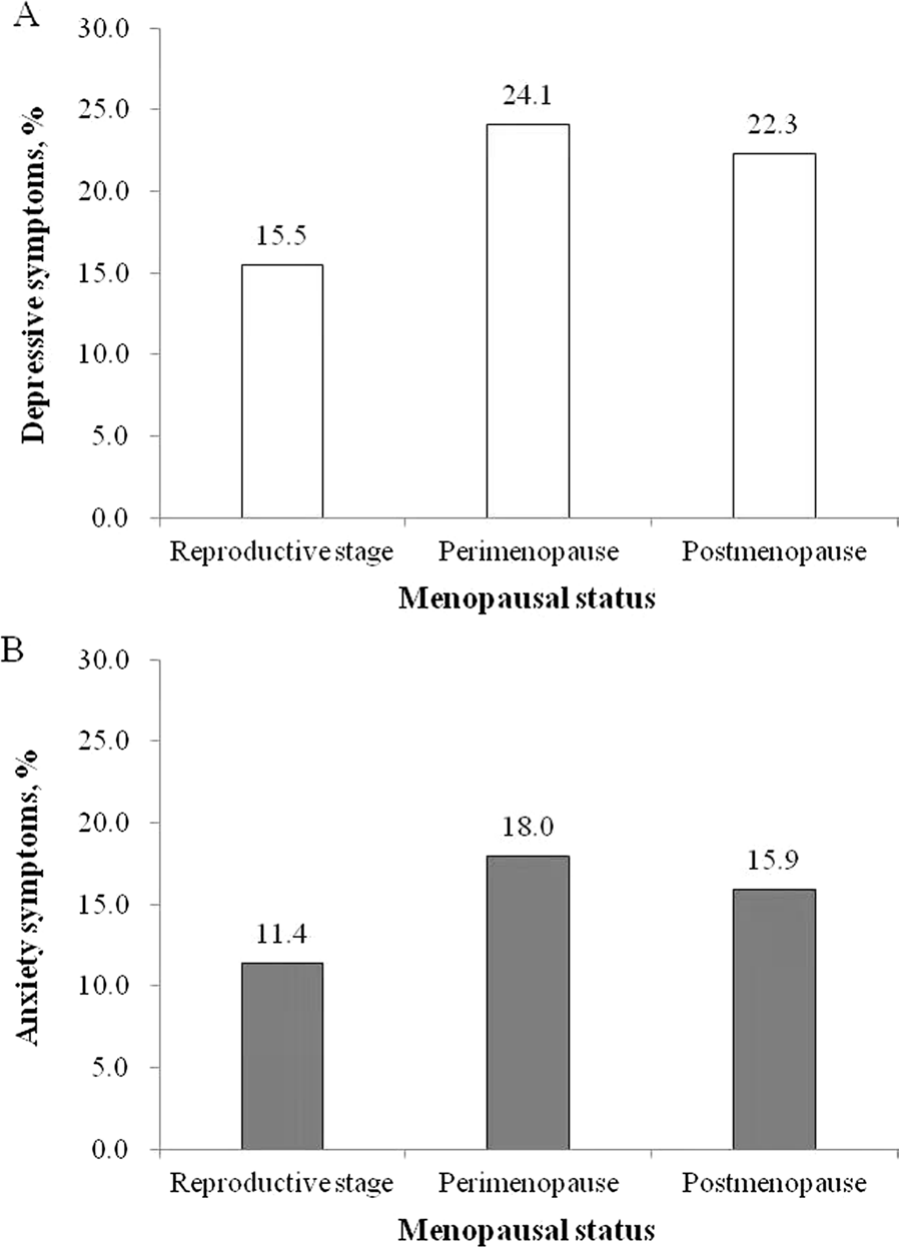
Percentage of depressive (**A**) and anxiety symptoms (**B**) by menopausal status

According to the PHQ-9 score, 19.5% (1 422/7 275) of participants experienced depressive symptoms, while 14.2% (1 035/7 275) of participants experienced anxiety symptoms based on the GAD-7 score. The prevalence of depressive symptoms was 15.5% (506/3 270), 24.1% (303/1 257) and 22.3% (613/2 748) in the reproductive stage, perimenopause and postmenopausal stage, respectively (Fig. 1A), whereas the rates of anxiety symptoms was 11.4% (373/3 270), 18.0% (226/1 257) and 15.9% (436/2 748) in the reproductive stage, perimenopause and postmenopausal stage, respectively (Fig. 1B).

### Associated factors of depressive symptoms

The univariate analysis revealed that age, place of residence, marital status, average monthly household income, smoking, regular physical activity, chronic diseases, menopausal status, vasomotor, somatic and urogenital symptoms showed statistically significant differences between women with and without depressive symptoms (all *P* < 0.05, Table 2). A multivariable logistic regression to evaluate independent associated factors for menopausal symptoms using potential variables (*P* < 0.1 in each univariate model) were shown in Table 3. Greater age (OR = 0.96, 95% CI: 0.95–0.98), higher levels of household income (5000–10,000 RMB: OR = 0.74, 95% CI: 0.64–0.86; > 10,000 RMB: OR = 0.54, 95% CI: 0.37–0.79), and regular physical activity (OR = 0.61, 95% CI: 0.53–0.69) were protective factors for depressive symptoms, while chronic diseases (OR = 1.29, 95% CI: 1.11–1.50), perimenopause (OR = 1.21, 95% CI: 1.01–1.45), vasomotor symptoms (OR = 1.43, 95% CI: 1.25–1.61), somatic symptoms (OR = 3.34, 95% CI: 2.89–3.86) and urogenital symptoms (OR = 2.48, 95% CI: 2.15–2.86) were found to be risk factors for depressive symptoms.

**Table 2:** Univariate analysis of independent variable on depressive and anxiety symptoms

	Depressive symptoms		*P* value	Anxiety symptoms		*P* value
	No (n = 5853)	Yes (n = 1422)		No (n = 6240)	Yes (n = 1035)	
*Sociodemographic characteristics*						
Age, years, median (IQR)	47 (44, 52)	48 (44, 53)	0.005	48 (44, 52)	48 (44, 53)	0.126
Place of residence			< 0.001			< 0.001
Urban	3016 (51.5)	642 (45.1)		3222 (51.6)	436 (42.1)	
Rural	2837 (48.5)	780 (54.9)		3018 (48.4)	599 (57.9)	
Education			0.694			0.691
High school and below	5298 (90.5)	1292 (90.9)		5649 (90.5)	941 (90.9)	
College or graduate school	555 (9.5)	130 (9.1)		591 (9.5)	94 (9.1)	
Employment status			0.359			0.114
Unemployed/retired	1141 (19.5)	262 (18.4)		1222 (19.6)	181 (17.5)	
Employed	4712 (80.5)	1160 (81.6)		5018 (80.4)	854 (82.5)	
Marital status			0.013			0.420
Married	5388 (92.1)	1280 (90.0)		5726 (91.8)	942 (91.0)	
Single/divorced/widowed	465 (7.9)	142 (10.0)		514 (8.2)	93 (9.0)	
Average monthly household income, RMB			< 0.001			< 0.001
< 5000	3802 (65.0)	1063 (74.8)		4092 (65.6)	773 (74.7)	
5000–10,000	1791 (30.6)	323 (22.7)		1886 (30.2)	228 (22.0)	
> 10,000	260 (4.4)	36 (2.5)		262 (4.2)	34 (3.3)	
Parity			0.134			0.218
Nulliparous	101 (1.7)	33 (2.3)		110 (1.8)	24 (2.3)	
Multiparous	5752 (98.3)	1389 (97.7)		6130 (98.2)	1011 (97.7)	
*Lifestyle factors*						
Drinking			0.383			0.140
No	5213 (89.1)	1255 (88.3)		5534 (88.7)	934 (90.2)	
Yes	640 (10.9)	167 (11.7)		706 (11.3)	101 (9.8)	
Smoking			0.033			0.855
Not current smoker	5707 (97.5)	1372 (96.5)		6071 (97.3)	1008 (97.4)	
Current smoker	146 (2.5)	50 (3.5)		169 (2.7)	27 (2.6)	
Regular physical activity			< 0.001			< 0.001
No	3377 (57.7)	972 (68.4)		3647 (58.5)	702 (67.8)	
Yes	2476 (42.3)	450 (31.6)		2593 (41.5)	333 (32.2)	
*Physical health factors*						
Body mass index, kg/m^2^			0.012			0.104
< 18.5	519 (8.9)	162 (11.4)		563 (9.0)	118 (11.4)	
18.5–23.9	3140 (53.6)	725 (51.0)		3334 (53.4)	531 (51.3)	
24–27.9	1651 (28.2)	417 (29.3)		1774 (28.4)	294 (28.4)	
≥ 28	543 (9.3)	118 (8.3)		569 (9.1)	92 (8.9)	
Chronic diseases			< 0.001			< 0.001
No	4798 (82.0)	1030 (72.4)		5077 (81.4)	751 (72.6)	
Yes	1055 (18.0)	392 (27.6)		1163 (18.6)	284 (27.4)	
*Menopausal factors*						
Menopausal status			< 0.001			< 0.001
Reproductive stage	2764 (47.2)	506 (35.6)		2897 (46.4)	373 (36.0)	
Perimenopause	954 (16.3)	303 (21.3)		1031 (16.5)	226 (21.8)	
Postmenopause	2135 (36.5)	613 (43.1)		2312 (37.1)	436 (42.1)	
Vasomotor symptoms			< 0.001			< 0.001
No	4346 (74.2)	747 (52.5)		4593 (73.6)	519 (50.1)	
Yes	1507 (25.8)	675 (47.5)		1647 (26.4)	516 (49.9)	
Somatic symptoms			< 0.001			< 0.001
No	4172 (71.3)	662 (46.5)		4649 (74.5)	365 (35.3)	
Yes	1681 (28.7)	760 (53.5)		1591 (25.5)	670 (64.7)	
Urogenital symptoms			< 0.001			< 0.001
No	5435 (92.9)	1020 (71.7)		3176 (50.9)	265 (25.6)	
Yes	418 (7.1)	402 (28.3)		3064 (49.1)	770 (74.4)	

Values are median (interquartile range) or n (%)

**Table 3:** Multivariable logistic regression analysis of related factors of depressive and anxiety symptoms

	Depressive symptoms		Anxiety symptoms	
	OR (95% CI)	*P* value	OR (95% CI)	*P* value
Age, years, median (IQR)	0.96 (0.95, 0.98)	< 0.001	–	–
Place of residence				
Urban	Ref		Ref	
Rural	1.03 (0.90, 1.17)	0.658	1.21 (1.04, 1.39)	0.011
Marital status				
Married	Ref			
Single/divorced/widowed	1.17 (0.94, 1.46)	0.157	–	–
Average monthly household income, RMB				
< 5000	Ref		Ref	
5000–10,000	0.74 (0.64, 0.86)	< 0.001	0.84 (0.71, 1.00)	0.054
> 10,000	0.54 (0.37, 0.79)	0.001	0.94 (0.64, 1.40)	0.772
Smoking				
Not current smoker	Ref			
Current smoker	1.27 (0.89, 1.82)	0.192	–	–
Regular physical activity				
No	Ref		Ref	
Yes	0.61 (0.53, 0.69)	< 0.001	0.67 (0.57, 0.78)	< 0.001
Body mass index, kg/m^2^				
< 18.5	0.99 (0.79, 1.23)	0.901	–	–
18.5–23.9	Ref			
24–27.9	0.97 (0.84, 1.13)	0.727	–	–
≥ 28	0.82 (0.65, 1.04)	0.098	–	–
Chronic diseases				
No	Ref		Ref	
Yes	1.29 (1.11, 1.50)	0.001	1.22 (1.03, 1.44)	0.021
Menopausal status				
Reproductive stage	Ref		Ref	
Perimenopause	1.21 (1.01, 1.45)	0.038	1.12 (0.92, 1.37)	0.245
Postmenopause	1.19 (0.97, 1.45)	0.094	0.89 (0.75, 1.05)	0.155
Vasomotor symptoms				
No	Ref		Ref	
Yes	1.43 (1.25, 1.61)	< 0.001	1.60 (1.38, 1.87)	< 0.001
Somatic symptoms				
No	Ref		Ref	
Yes	3.34 (2.89, 3.86)	< 0.001	3.62 (3.08, 4.25)	< 0.001
Urogenital symptoms				
No	Ref		Ref	
Yes	2.48 (2.15, 2.86)	< 0.001	1.53 (1.29, 1.82)	< 0.001

### Associated factors of anxiety symptoms

The univariate analysis demonstrated that place of residence, average monthly household income, regular physical activity, chronic diseases, menopausal status, vasomotor, somatic and urogenital symptoms had statistically significant differences between women with and without anxiety symptoms (all *P* < 0.05, Table 2). In the multivariable model, place of residence, regular physical activity, chronic diseases, vasomotor, somatic and urogenital symptoms were independently associated with the risk of anxiety symptoms (all *P* < 0.05, Table 3).

## Discussion

In this large-scale cross-sectional study of Chinese women aged 40–60 years, 19.5% and 14.2% of women reported to experience depressive and anxiety symptoms, respectively. In the reproductive stage, perimenopausal and postmenopausal stage, the rates of depressive symptoms were 15.5%, 24.1% and 22.3%, respectively, while the prevalence of anxiety symptoms were 11.4%, 18.0% and 15.9%, respectively. In addition, we also found that age, average monthly household income, regular physical activity, chronic diseases, menopausal status, vasomotor symptoms, somatic symptoms and urogenital symptoms were associated with the risk of depressive symptoms, whereas place of residence, regular physical activity, chronic diseases, vasomotor symptoms, somatic symptoms and urogenital symptoms were associated with the risk of anxiety symptoms.

In this study, 19.5% of women experienced depressive symptoms assessed by the PHQ-9, while 14.2% of participants reported having anxiety symptoms based on the GAD-7. Several earlier epidemiologic studies conducted in different areas of China also examined the prevalence of depressive and anxiety symptoms with dissimilar results [5, 36, 37]. A previous cross-sectional study involving 1,062 middle-aged women in Shanghai, an eastern region of China, used the self-rating depression syndrome and the self-rating anxiety syndrome to measure symptoms of depression and anxiety, respectively, and reported a prevalence of depressive and anxiety was 26.0% and 12.6%, respectively [5]. Another longitudinal study performed in 430 women from Beijing located in Northern China indicated that 18.2% and 7.0% of middle-aged women during the menopausal transition suffered from depressive and anxiety symptoms [36]. Furthermore, a recent meta-analysis of observational studies revealed that the prevalence of depressive symptoms in menopausal Chinese women was 36.3%, ranging from 22.3% in the western region to 42.3% in the northeast region [37]. These inconsistent results might be partly owing to regional disparity, discrepancy in the study sample size, and the utilization of different measurements of depressive and anxiety symptoms.

This study found that the prevalence of depressive symptoms were 15.5%, 24.1% and 22.3% in the reproductive stage, perimenopausal and postmenopausal stage, respectively. Consistent to our findings, a cross-sectional study conducted in the Taiwanese community also suggested that perimenopausal women (23.3%) were more likely to suffer from depressive symptoms than postmenopausal women (17.2%) [38]. In contrary to our findings, a cross-sectional survey involving rural Chinese midlife women revealed that postmenopausal women had the highest prevalence of depressive symptoms (17.25%) than premenopausal (6.89%) and perimenopausal women (9.6%) [39]. Additionally, we also observed that menopausal status was associated with higher risk of depressive symptoms after adjustment for potential confounders. Consistent with our findings, a previous meta-analysis based on 11 studies also demonstrated that women in perimenopause had a twofold increased risk of depressive symptoms than those in the premenopausal stage [40]. The aforementioned cross-sectional, population-based study including 3,359 midlife Taiwanese women also indicated that perimenopause was significantly associated with the risk of depressive symptoms [38]. Furthermore, a prospective study involving 711 middle-aged Australian women revealed that menopausal status was associated with the risk of depressive symptoms, and found a greater likelihood of elevated depressive symptoms during perimenopause [41]. Moreover, this study also observed that the prevalence of anxiety symptoms were 11.4%, 18.0% and 15.9% in the reproductive stage, perimenopausal and postmenopausal stage, respectively. In line with our findings, previous studies also indicated that perimenopausal women had the highest prevalence of anxiety symptoms (17.7%) according to the hospital anxiety and depression scale, followed by 17.5% of postmenopausal women and 14.4 of perimenopausal women [42]. However, compared to the depressive symptoms, anxiety symptoms among middle-aged women have drawn relatively little attention. Further large-scale longitudinal studies are needed to describe the change in the rates of anxiety symptoms according to different menopausal status, as well as explore the association between menopausal status and anxiety symptoms.

This study demonstrated that age was associated with depressive symptoms. Consistent with our findings, evidence based Chinese and American populations also reported a negative correlation between age and depressive symptoms among middle-aged women [43, 44], which might be partly interpreted by that women might be able to endure more stress of job, family and life events as age increased and subsequent reducing the risk of depressive symptoms [43]. Furthermore, we also found that family income was negatively associated with depressive symptoms, which was in concordance with earlier studies based on Chinese and Turkish populations reporting that women with lower levels of family income were susceptible to having depressive symptoms [43, 45–47]. Additionally, our findings also indicated that women living in rural areas were more likely to experience anxiety symptoms compared to those living in urban areas, which might be explained by the differences in social and personal attitude towards aging and menopause, knowledge of menopausal symptoms, and awareness of treatment for menopausal symptoms between women living in rural and urban areas [48].

Our findings may have public health implications, since these results suggest that regular physical activity might improve depressive and anxiety symptoms among middle-aged women. This study indicated that women who engaged in regular physical activity had 39% and 33% lower risk of depressive and anxiety symptoms, respectively, than those without regular physical activity. In parallel to our results, several previous studies of Iranian, American, Korean and Chinese populations also reported a negative association between regular physical activity and depressive symptoms [49–51]. Moreover, a randomized controlled trial showed that 12 weeks of moderate-intensity aerobic exercise slightly improved depressive and insomnia symptoms in midlife sedentary women [52]. Given the benefits of physical activity, health education should be provided to middle-aged women, and encourage them to engage in regular physical activity to improve their mental conditions.

One interesting finding of this study was that menopausal symptoms including vasomotor, somatic (including paresthesia, dizziness, arthralgia and myalgiam, headache, palpitations, skin formication) and urogenital symptoms were associated with the risk of depressive and anxiety symptoms. Several studies have revealed that vasomotor symptoms were associated with the risk of depressive symptoms in the perimenopausal and postmenopausal stages or throughout three menopausal statuses [39, 42, 53–55]. However, a prospective community-based cohort study including 430 urban Chinese women indicated that vasomotor symptoms were independently correlated with depressive symptoms but not with anxiety symptoms [13]. Consistent with our findings, another cross-sectional study conducted on 1,062 midlife Chinese women also used the KMI to measure menopausal syndrome and reported a positive correlation between menopausal syndrome and symptoms of depression and anxiety [5]. Moreover, a previous cross-sectional study of 1,280 Chinese women around menopause also showed that urogenital symptoms, such as dry vagina and dyspareunia, were related to symptoms of depression and anxiety [45]. The potential mechanisms underlying the link between menopausal symptoms and symptoms of depression and anxiety might partly due to the change in gonadal hormones, which was supported by the evidence indicating that hormonal change could contribute to depression in susceptible women [56]. In addition, previous study revealed that antidepressants were able to alleviate several menopausal symptoms including hot flashes and night sweats [57, 58].

The strengths of this study contain a large sample size, and the measurement of anxiety, depressive and menopausal symptoms using validated tools. This study also had several limitations. First, this study cannot evaluate the causality between depressive and anxiety symptoms and their associated factors because of the cross-sectional study design. Second, our data only included Chinese Han population, which limited the generalizability of the results to middle-aged women of other ethnic groups. Finally, we did not collect the data on the use of anti-depressant and anti-anxiety medication, so we unable to evaluate the influence of the use of anti-depressant and anti-anxiety medication on depressive and anxiety symptoms among middle-aged women.

## Conclusions

In conclusion, our study found that middle-aged women with chronic diseases, during perimenopause, and having menopausal symptoms had a greater likelihood of depressive symptoms, while women with greater age, higher levels of family income, and regular physical activity were less likely to experience depressive symptoms. In addition, women living in rural areas, having chronic diseases and menopausal symptoms, and who did not engage in regular physical activity were associated with increased risk of anxiety symptoms. Further research is needed to confirm our findings and to elucidate the potential mechanisms underlying the relationship between symptoms of depressive and anxiety and their related factors in middle-aged women.

## Data Availability

The datasets used during the current study are not publicly available due to the privacy policy, but are available from the corresponding author on reasonable request.

## Abbreviations

BMI: Body mass index
GAD-7: Generalized anxiety disorder-7
KMI: Kupperman menopausal index
PHQ-9: Patient health questionnaire-9
